# The Andrew Clark Memorial

**Published:** 1895-12-28

**Authors:** 


### The Hospital

An Institutional Journal of

The Medical Sciences and Hospital Administration, with

AN EXTRA NURSING SUPPLEMENT.

Vol. XIX.—No. 483.] [Dec. 28, 1895.

### THE ANDREW CLARK MEMORIAL.

With intense satisfaction we have to announce that the proposal to perpetuate the memory of Sir Andrew Clark by the erection of an "erysipelas ward for women, another for isolation cases, and increased accommodation for the porters at London Hospital" has been abandoned. The credit for this wise decision must be shared between the honorary medical staff and the House Committee of the London Hospital. When the meeting of the Andrew Clark Memorial Committee was held about a month ago to consider the matter, a report was submitted which announced that the form of memorial approved by the governors of the London Hospital at the quarterly court in September on the recommendation of the House Committee had been declared by the staff to be one which they could not support. It consequently fell to the ground. After full discussion it was determined to communicate with the committee of the London Hospital and with the council of the medical college attached to it, with the object of obtaining definite information on two points: First, as to whether the House Committee would undertake to invest the funds available for this memorial pending their being in a position to add to them a sufficient sum to carry out the original proposals which involved an outlay of £12,000, and, if so, whether the House Committee would consent to fix a limited period in which this work should be undertaken? If it was not so undertaken at the expiration of the time fixed, then it was asked if the House Committee would return the money with interest to the Memorial Committee to be devoted to some other form of memorial? Secondly, the Council of the London Hospital Medical College were asked to report if there were any objects connected with the school which could be completed for the sum now available, *i.e.*, £2,500, after the payment of expenses and excluding the moneys voted by the London Hospital Committee, amounting to £2,000 more?

The reports from the House Committee and from the Medical College Council were presented to a meeting of the Memorial Committee on Thursday last. The Council stated that they could suggest three objects to which the money might be usefully devoted as a memorial to Sir Andrew Clark, providing the House Committee felt in all the circumstances that they could not see their way to erect an Andrew Clark Memorial Wing, as originally planned. The House Committee reported that they were prepared to receive the £2,500 in trust to invest it as the nucleus of a building fund, and to use their best endeavours to raise that fund to £12,000, and to erect the Andrew Clark Memorial Wing within five years from the present date. If for any cause they should find it impossible to erect this wing, then the House Committee would return the money to the Memorial Committee, leaving them to expend it on such other object as they might select for the memorial. We publish an account of the meeting referred to on another page of the present issue.

It is a matter for congratulation that the scheme carried at the quarterly court of the governors of the London Hospital in September last has been abandoned. We felt called upon to raise the strongest protest at the time against it, and we have to acknowledge with warm appreciation the support extended to our protest by the *Times*, which has no doubt contributed largely to secure the wise solution which has now been arrived at. The vigour of our protest caused, we fear, some annoyance to several good friends who are actively engaged in the management of the London Hospital.

When we wrote this protest we understood the governors' decision was final, and that there was no other appeal except to public opinion. We are free to confess, had we understood that the scheme of the House Committee was subject to ratification by the Memorial Committee before it could be carried out, we should probably have held our hand pending the decision of the latter committee. No individual, and certainly no organ of public opinion, however, can be properly regarded as a true friend unless candid criticism is sometimes indulged in to prevent errors, which may be, and often are, followed by grievous mischief even to the most worthy of men and institutions. We have always upheld the authorities of the London Hospital when we have felt them to be in the right, and we recognise with warm appreciation the steps they have now taken to remedy an initial mistake, which had its origin, no doubt, in the best intentions, though the results likely to accrue were not fully appreciated by those who were responsible for the decision.

It affords us real pleasure to testify to the handsome conduct of the House Committee of the London Hospital, and especially of its chairman (Mr. Hale) and its treasurer (Mr. J. H. Buxton), who both devote much time and energy to the interests of the institution which they serve so well. The honorary medical staff, too, deserve praise for the wisdom they displayed in withholding their sanction to the scheme which we felt it our duty to resist to the utmost. It is satisfactory to know that the solution now arrived at has been unanimously approved by everyone interested in the Andrew Clark Memorial, that it is worthy of the wisdom which ought to guide the affairs of so great an institution as the London Hospital, and that it will, we have no doubt, ultimately result in the erection of an Andrew Clark Memorial Wing worthy of the man, his reputation, and the life's work which he gave to the great hospital with which his name will be for ever connected.

We are confident that the happy results which have attended the recent discussions upon this memorial will restore public confidence in the management of the London Hospital and tend to prevent hasty and inadequate proposals from finding acceptance in the future, should occasion arise to raise a monument to the memory of any man who, like the late Sir Andrew Clark, is deemed worthy of lasting public recognition. As the *Times* said on a recent occasion: "Not our bridges, or railways, or telephones, but the great fortresses of science and benevolence, erected where suffering most abounds, are the real glory and abiding distinctions of our civilisation." There is all the more reason then that the men who have erected, or whose life's work has materially tended to strengthen and maintain, these great fortresses of civilisation, should first be memorialised by their works which follow them.

